# Can Animals Be the Key to the Development of Tourism: A Case Study of Livestock in Agritourism

**DOI:** 10.3390/ani11082357

**Published:** 2021-08-09

**Authors:** Anna Jęczmyk, Jarosław Uglis, Ryszard Steppa

**Affiliations:** Laboratory of Rural Tourism, Department of Zoology, Faculty of Veterinary Medicine and Animal Science, Poznan University of Life Sciences, Wojska Polskiego Street 28, 60-637 Poznan, Poland; jaroslaw.uglis@up.poznan.pl (J.U.); ryszard.steppa@up.poznan.pl (R.S.)

**Keywords:** farm animals, livestock, animal welfare, animal use, tourist attraction, tourist experience, agritourism

## Abstract

**Simple Summary:**

Animals are an integral and important part of human life. It is clear that when traveling, tourists often choose places where animals are present. They look for places with wild animals more often. Our article introduces issues concerning the role of farm animals in the creation of agritourism products and the prospects of using animals in agritourism. On the one hand, farm animals are an additional source for people who run agritourism farms and make the stay of tourists in rural areas more attractive. The presence of livestock acts as a magnet, attracting more tourists to these farms and achieving higher returns on agritourism activities. However, among the reasons for the lack of contact with animals in agritourism offerings, farmers indicated: the lack of financial resources and the lack of conditions to provide the animals with adequate maintenance. It is worth introducing farm animals as a tourist attraction in agritourism offerings.

**Abstract:**

Animals are an integral part of human life. Currently, they constitute a relevant factor contributing to the tourist experiences of individuals travelling for tourist purposes. Domestic (farm) animals constitute a tourist attraction, are a source of income for individuals running agritourism farms, and render the tourists’ stay in rural areas more attractive. It is important to maintain the welfare of livestock based on principles of sustainability. The authors conducted research among agritourism providers in Wielkopolska Voivodeship. The aim of the survey was to determine whether agritourism farms keep livestock, what species they are, and whether they are important in building an agritourism product and what the prospects are for using animals in agritourism. A questionnaire was used for the study. The use of a variety of animal species facilitates the maintenance of biodiversity on the farm. Our research shows that 57.3% of the analysed agritourism providers included animals. Additionally, we discovered that the presence of livestock acts as a magnet that draws more tourists towards the individuals running agritourism farms and thus causes them to obtain higher profit from the agritourism business. According to the surveyed individuals, the most influential reason for the absence of livestock on their farms was the lack of financial resources and conditions to provide the animals with proper maintenance. Such findings may prompt the introduction of livestock as a tourist attraction in agritourism in other regions.

## 1. Introduction

Animals have been accompanying humans for centuries [1], constituting an essential part of the surrounding ecosystem [2]. They interact with humans on the level of production and coexistence. In this case, production should be understood as the provision of food and animal raw materials, such as clothing [3]. Keeping livestock for food purposes fits perfectly into this notion. In turn, coexistence refers to wild animals whose presence is important for maintaining biodiversity in the ecosystem.

Animals are an important and influential element of tourism. For many people, they constitute an integral part of their valued recreational activities [4]. Tourism provides many people with the opportunity to see and interact with a variety of species, which they would not normally encounter, all over the world and often in their natural habitat [5].

According to World Animal Protection, more than half a million wild animals are used as tourist entertainment, yet this number includes only the registered animals in tourist facilities [6]. Wildlife tourism is defined as tourism based on encounters with non-domesticated (non-human) animals and may be divided into non-consumptive and consumptive. The former involves observing or interacting with animals in natural or man-made environments, while consumptive wildlife tourism entails the intentional killing of animals, usually through hunting or fishing [7]. 

Non-consumptive wildlife tourism accounts for 20 to 40% of all international tourism [8]. It is estimated that between 230,000 and 550,000 animals are currently used for recreational purposes, and only 1500 to 13,000 animals in the tourism industry are treated in a way that ensures their good condition and safety [9]. An analysis of comments concerning animal-related attractions posted by tourists on TripAdvisor reveals that 80% of travellers do not recognise the problem or take into consideration animal welfare [10].

Wildlife tourism provides nature with the opportunity to earn its own right to survive [11], while the economic benefits it generates serve to protect wild animals and their natural habitat [12]. Consistently high demand for encounters with captive, partially captive, and wild animals persists [13], and the funds raised in this regard generate profit for communities and activities focused on the conservation of nature around the world [14].

Tourism uses various species of animals in different ways. Opportunities to observe or interact with wild animals have been created in numerous tourist destinations that are easily accessible and very popular among many consumers. Such opportunities vary from country to country, with each destination having its own legal and cultural approach to animal welfare. Animals can participate in festivals, be used as street entertainment, or be observed in captivity. They are also frequently associated with the source of income of local communities [15]. Tourists decide to travel to see and interact (pet, swim with, ride, take selfies) with dolphins, tigers, and other charismatic, endangered, and exotic animals [16]. The tourism industry includes a variety of attractions for tourists to participate in: from safaris providing the opportunity to observe wild animals, zoos, meeting and cuddling cats in cafes, or volunteering to work with goats on farms, to “danger tourism” involving close encounters with fearsome predators [17].

The creation of new tourism products is an ongoing process. One of its primary issues consists of creating them in a way that allows attracting the largest possible number of tourists. Regardless of the geographical region or wildlife, observing, photographing, or having direct contact with wild animals has always been the aim of travels for individuals who value interactions with animals. An example may be trips to Africa organised to observe the so-called “Africa’s big five animals” [18], gorillas [19], and cetaceans [20] and to participate in birdwatching, which is becoming increasingly popular throughout the world [21]. Tourists are capable of negatively affecting animal welfare, as evidenced in the study by Moorhouse et al. [10]. Moreover, it should be noted that wild animals may pose a threat to tourists—such examples include brown bear [22] and polar bear attacks [23]. Fishing [24] and hunting tourism, which, contrary to numerous opinions, can contribute to the protection of many species, is also very popular [25]. A certain form of interaction with animals is also culinary tourism, which manifests by the desire to taste food prepared from a particular species of animal in the place of its occurrence. A very popular example involves the consumption of seafood in coastal areas.

Categories of wild and domestic animals are not fixed and permanent—they are created by humans and contain numerous contradictions [26]. Animals are part of human life in many ways: from pets and farm animals to wildlife. Humans have used them as a food source, labour force, companions, or simply products for centuries. For most of human history, animals have been used for their utility value. Animals and animal products have been directly used to satisfy the needs of individuals or entire communities [12]. They should not be regarded as commodities that may be utilised for the benefit of humans [27]. 

Changes in tourists’ tastes may be used to improve standards of animal welfare in the future. Moreover, many Western tourists are inclined to practise “sustainable”, “eco”, and “ethical” tourism [17].

Currently, more attention is focused on the welfare of kept animals. For many modern societies, this is an issue of increasing importance, which dates back to the early British animal protection laws of the 19th century [28]. A proper approach to the welfare of livestock has created, at least in the case of some consumers, both the desire and demand for products derived from animals raised following welfare standards but has also satisfied that demand through the development of a supply chain involving such products. That is precisely how animal welfare transformed into a distinctive product value [29]. Furthermore, there is growing interest in animal welfare in a range of tourism recreation contexts also with regard to tourist activity [30]. 

Research on domestic animals in tourism, including livestock, has received much less attention than studies on wild animals [16]. Four reasons responsible for this state of affairs can be identified [31]:

1. Unequal power relations, according to which humans control all aspects of animals’ lives;
2. Domestic animals are “everyday” animals, which indicates they are not regarded with the kind of interest that tourists take in strange and exotic animals;
3. There is tension created by a significant number of domestic animals used as a source of food;
4. Due to their controlled existence, domestic animals are perceived as less authentic than wild animals.

Undoubtedly, human–animal interactions are extremely important components of the overall experience for humans [32]. Animals are an integral element of countless human leisure experiences and positively affect their, i.e., humans’, physical, mental, and social condition [33]. The therapeutic role of animals in leisure time is widely recognised [5] and includes examples such as hippotherapy [34], dog therapy [35], feline therapy [36], donkey therapy [37], and apitherapy [38].

One form of tourism in which animals, particularly livestock, are part of a commercial product directed at tourists is agritourism. The literature on the subject does not include works that focus on the use of domestic animals in tourism. Our work helps to fill this gap.

Livestock can be a potentially profitable enterprise for small-scale agricultural operations. Livestock can offer a farm new revenue streams as well as increased fertility and weed control. Additionally, this activity can become a tourist attraction and can attract tourists, giving the farm a new function, a tourist function. However, there are new challenges of raising livestock on agritourism farms which are discussed here, including particular considerations related to producing poultry, rabbits, hogs, sheep, goats, bees, and cattle. 

This study aims to determine whether farm animals—and if so, which species—are kept on agritourism farms, and whether the farm animals affect the attractiveness of the agritourism offering.

### Literature Review

Agritourism is not a new phenomenon. Busby and Rendle [39] believe that since the beginning of the 20th century, farmers have expanded recreational opportunities (that they offered) to include accommodation services. At first, agritourism was regarded as an additional, secondary activity performed on a farm but then quickly gained popularity and became one of the forms of tourism [40]. It was a universal means to mitigate problems at the farm level as a strategy for economic growth and diversification [41], but also at the regional and local level of rural areas, i.e., migration [42] or affecting the increase in income of the local community [43]. Agritourism allowed for acquiring additional benefits at the regional level, i.e., creation and maintenance of jobs, environmental improvement in rural regions, and rural heritage and identity preservation [44]. However, the agritourism relationship between agriculture and tourism is symbiotic [45,46]. Agritourism is a practice of attracting tourists to agricultural areas, generally for educational or recreational purposes. Due to the economic difficulties, as well as changes in agriculture and animal husbandry around the world, many farmers—particularly those running small family farms—found that they need to expand their agricultural business model and discover new methods of generating income.

The most important specific features of agritourism distinguishing it from other forms of tourism are the elements that connect the main agricultural and tourist activities, which are the rhythm of life on the farm, the presence of livestock, fresh food, smells, sounds, the proximity of the farming family and villagers, possibility of getting to know family habits, everyday activities of villagers, hospitality, new acquaintances and friendships, culture and customs, tradition and history of villages and the region, folklore, space, contact with nature, freedom of movement, silence, peace, possibility of recreation and sports, etc. [47,48,49]. Undoubtedly, an important element of agritourism is the accommodation of tourists in the house of a farmer offering agritourism services [43,50]. Adapting a residential house (furnishing rooms, etc.) and its immediate surroundings for guests (e.g., a recreational device) is associated with getting to know the needs and expectations of potential agritourists [51].

Majewski [48] emphasises that agritourism should be conducted on a farm, where plant and animal production constitutes one of the biggest attractions for tourists. Currently, the concept of agritourism [52] includes resting at the facilities maintained by the farmer and located on a functioning farm, where apart from spending the night, eating meals made of products derived from the farm or its vicinity, participating in field and farm work, and observing farm animals and plant production, it is also possible to undertake recreational activities on the area of the farm and beyond. Authentic agritourism is conducted on a fully operational farm, where agricultural activities predominate over tourism, as well as where familiar and direct contact with the host and his or her family members takes place in an unaltered agricultural environment [53]. A farm on which the farmer keeps various species and breeds of animals is more attractive to visitors. This involves the contact of tourists, mainly children, with live animals, such as poultry, rabbits, goats, sheep, calves, etc. [49].

Agritourism shows people what is happening on the farm in order to better inform them about daily agricultural practices and activities. Its underlying assumption is that if people see the farm, they will better understand what is happening there and why, which in turn should reduce concerns about animal welfare or production practices [54]. Farm and farm-related activities can be divided into five groups as follows:

− Observation of the agricultural production process including plant and animal production and food processing, as well as guided tours or private farms (ranches), which are widely offered in different countries;
− Actual participation in the process of plant and animal production and processing (e.g., assistance in milking cows, hay production, etc.);
− Animal shows or demonstrations, including cow milking, sheep shearing, angora rabbit shearing, cattle sales, or cowboy rodeos;
− Marked trails on the farm;
− Direct contact with domestic animals or the nature of farms in different kinds of petting zoos or safaris [49].

Farm animals may constitute a valuable agritourism attraction while simultaneously being part of the cultural heritage of rural areas [55]. For many small-scale livestock farmers, species and breeds of these animals are linked to unique domestic systems of knowledge and management practices, which have been gathered over centuries by countless generations [56].

To be part of the tourism product and a tourist attraction, animals must be provided with adequate welfare which is not necessarily better on small farms compared to large farms. Observing the behaviour of farm animals is the first element in assessing their physical and mental comfort [57].

Freedom to perform natural behaviour, however, is the main issue in the eyes of the general public, so technologies in which the use of enclosures and pastures that should be the basis of nutrition for many species during the growing season play a huge role. These technologies simultaneously ensure a better quality of food raw materials [58]. Keeping animals outside of buildings provides better opportunities for tourists to observe and interact with animals. Landscape conservation is also an important function of livestock grazing [59].

Another method includes using livestock as a raw material for meal preparation. The increasing tendency to seek locally produced food applies particularly to the hospitality and tourism industry, including agritourism, which heavily relies on local identity [60].

Aspects such as humane treatment of animals, the environmental impact of the main supply chain, and the interest of tourists in tasting local food, especially meat, in agritourism is important for many consumers [60]. Tourists seem to particularly favour culinary tourism [61], which is implemented on agritourism farms. Hall and Sharples [62] believe that culinary tourism is associated with unforgettable experiences related to food and drinks, as well as places where good food is prepared for entertainment purposes, but also includes visits to local producers, local markets, cooking shows, and all activities related to tourism and food [63]. This form of tourism allows for the use of resources from agritourism farms and their surroundings [64].

The agritourism product is a complex category, and its individual elements perform different functions [65]. Livestock can become a product with different purposes, i.e., for people from cities who do not have contact with animals, they can meet the need for interacting with animals, and for city children, they can meet the need to better understand animals and their role in, e.g., human nutrition. 

According to Sayre and Henderson [66], animals on a farm can be used in a variety of ways. It all depends both on the species that the agritourism operator has and on their creativity.

According to Hurst and Niehm [67], the market of local products in rural areas is largely untapped by both vacationers and residents. If the owners of agritourism farms were able to increase those sales, including meat produced based on principles of sustainability, not only would they raise their income, but they would also diversify their income sources [60].

## 2. Materials and Methods

The study was conducted in 2019, in the form of a telephone interview called CATI (computer assisted telephone interviews), based on questionnaires (Supplementary Materials) prepared by the authors of the paper. Telephone interviews are now widely used in scientific research [43,68,69,70].

The questionnaire consists of the following parts: part I—questions related to the location of the agritourism farm (3 questions), part II—records and a brief description of agritourism and agricultural activities (8 questions), part III—identification of species kept on the agritourism farm (2 questions), part IV—assessment of the attractiveness of animals kept on an agritourism farm (5 questions). Seven reasons for the lack of animals are identified in the questionnaire. At the end of the questionnaire, the respondents could indicate which species of animals, in their opinion, could make the agritourism offering more attractive.

Sample size determination is an important major step in the design of a research study. Its calculation is an essential step in research protocols and is a must to justify the size of research studies in papers, reports, etc. [71,72]. 

The study sample consisted of 394 agritourism farms from a total number of 686 farms (as of 2017) located in Wielkopolska Voivodeship. 

The finite population sample size formula was used for calculating the sample size of agritourism farms covered by this study:

(1) $$n=\frac{P\left(1-P\right)}{\frac{e^{2}}{Z_{\alpha /2}^{2}}+\frac{P\left(1-P\right)}{N}}$$

where *n* is sample size, *e* is permissible error, *N* is population size, and *Z*_*α*/2_ is value resulting from the confidence interval used; for a 95% confidence level, *Z*_*α*/2_ = 1.96, and *P* is the estimated proportion in the population (usually, it is set at *P* = 50%). *P* is the estimated expected proportion in the population covered by the study. As the proportion in the population of operators is unknown, the least favourable assumption was made, namely that *P* = 50%, because at that *P* level, the product *P*(1 − *P*) reaches the maximum value.

It is thus assumed that:

*P* = 50%;
Confidence level = 95%;
Permissible error = 5%;
*N* = 686 (the number of agritourism farms in 2017).

The minimum sample size was 246 when the maximum error was 5%. Participation in the study was voluntary and anonymous. When entering the study, it was assumed that if the telephone contact was not possible during the first phone attempt, the next phone attempt would be conducted after one hour. When the second phone attempt was not successful, subsequent phone attempts were made the next day. A total of 394 phone calls were obtained during the survey. All data gleaned were subjected to preliminary analysis, followed by statistical analysis, with the identification of significant differences for two independent samples by the Mann–Whitney U test and the Wald–Wolfowitz runs test. The statistical analysis was performed using the STATISTICA 13.3 program (TIBCO Software Inc., Palo Alto, CA, USA). Three data presentation methods were used to characterise the phenomena examined: tabular, graphical, and descriptive.

## 3. Results

### 3.1. Characteristics of Agritourism Activities in the Researched Agritourism Farms

A total of 365 providers of agritourism agreed to participate in the survey and be interviewed, while 29 refused to participate in the survey. According to the survey, 117 agritourism operators did not run any agritourism business in 2019. The agritourism activity related to hosting tourists on a working farm was carried out by 248 farmers. Only 142 farms out of all the analysed farms kept animals.

Given the period of conducting the agritourism business, it was found that the oldest agritourism farm hosted tourists continuously since 1980, while the youngest agritourism farm started hosting guests for on-farm recreation in 2018. It should be noted that more than 1/3 of the surveyed agritourism farms were established in the 20th century. 

The accommodation base is the most important component of agritourism. All the quarters included in the research provided their agritourists with accommodation in rooms. The number of rooms largely determines the possibility of accommodating a certain number of guests, and the number of beds is also important. The average number of rooms in the researched farms was up to five (80.6%), and the number of beds was 15 (Me = 12). Analysing the data on the number of tourists enjoying leisure in the surveyed agritourism farms, it ranged from 10 to 500 per year.

### 3.2. Characteristics of Kept Animals in the Researched Agritourism Farms

To achieve the research objective, the surveyed agritourism farms were divided into two groups. The first group consisted of providers of agritourism who owned livestock: 142 farms with animals. The second group included agritourism operators who did not own animals: 106 animal-free farms. The study is shows that 57.3% of the analysed agritourism farms in the Wielkopolskie Voivodeship featured animals.

When analysing the data concerning agricultural activities carried out in the surveyed farms (Table 1), statistically significant differentiation was found in terms of the agricultural holding area. Undoubtedly, livestock husbandry is associated with having adequate space for these animals. The main purpose of livestock husbandry was a sale, while the secondary purpose was an attraction for tourists who enjoy spending holidays in the countryside. This is confirmed by the share of agricultural income in total income, which was higher for agritourism operators who owned animals than those who did not own animals. Moreover, a significantly higher share of other income sources (67.9%) was found for providers of agritourism who did not own animals.

On the other hand, taking into account the share of income from agritourism activity, a statistically significantly higher level was found among operators with animals. Higher income from agritourism activities in facilities with animals resulted from the greater number of tourists using their offering. Their services were used on average by 117 tourists, and the average for operators without animals was 73 tourists. It is worth emphasizing that the average value of the number of tourists turned out to be statistically significant (*p* < 0.000). There was no statistically significant differentiation (*p* > 0.05) in the number of rooms and number of beds between the studied groups

Agritourism operators usually keep several species of animals on their farm. Horses (52.8%), hens (62.7%), cattle (18.3%), goats (16.9%), sheep (12.7%) and pigs (12.7%) were the most popular animals kept on farms. It should be noted that one in five of the surveyed facilities kept fish in ponds owned by the farm, and one in six of the surveyed facilities kept rabbits. 

Livestock can become an element of the core of an agritourism product, a basic product, or an extended product (Table 2). For people from cities who do not have contact with animals, they can meet the need for interacting with animals, and for city children, they can meet the need to better understand animals and their role in, e.g., human nutrition. As a basic product, they can be one of the components of the diet of tourists staying at an agritourism farm or culinary products prepared for sale. Being an element of an extended product, which distinguishes a given offer from other competitors, they can give the opportunity to learn how to prepare a local culinary product, participate in the handling of animals, or even organize a mini zoo and use the healing properties of contact with animals as part of animal therapy.

### 3.3. Reasons for Keeping and Lack of Animals in the Agritourism Farms

The choice to keep animals is not accidental since these animals are the key to increasing the tourist attractiveness of the run business. They are the source of meat, excluding horses, which are not eaten in Poland, and they are the source of valued products such as milk, eggs, and fish. The conducted analyses also showed that during the selection of animal species, agritourism operators paid attention to their origin and importance for the conservation of rural heritage. Therefore, they most often chose native breeds of livestock. 

**Figure 1:**
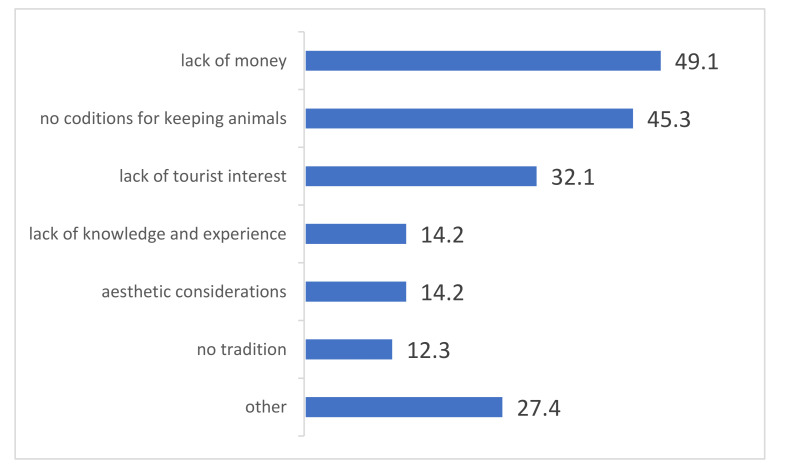
Reasons for not keeping livestock. Source: own studies.

The respondents who did not keep animals were further asked about the reasons for not keeping animals on the farm (Figure 1). 

According to almost half (49.1%) of the providers of agritourism, lack of money to keep animals, followed by lack of conditions to keep them (45.3%), is the main reason for not having animals. Interestingly, according to one third of the respondents, the reason for the absence of animals in their agritourism farm is the lack of interest from tourists.

First of all, the respondents indicated the reasons that were included in the answer to the questionnaire question; in addition, some of them gave other reasons that were not on the list of answers to the question.

One of the reasons for not keeping animals on an agritourism farm was the poor health and age of the farm owners.

*Respondent 1: “My age and health condition do not allow me to take care of animals”.*

Many owners indicated that the reason for the lack of animals on the agritourism farm was the lack of time but also the lack of people who would be able to help with the care of animals.

*Respondent 2: “I don’t have time to work with animals; I miss extra hands to work with animals*”.

Several respondents indicated that the reason for the lack of keeping animals on an agritourism farm was the lack of appropriate infrastructure, and more specifically of buildings in which these animals can be kept.

*Respondent 3: “I don’t have suitable buildings for keeping animals”.*

In contrast to several owners of the surveyed agritourism farms, some indicated that the reason for not keeping animals on their farm was cooperation with other farmers who had animals, and tourists could see them there.

*Respondent 4: “The animals are at my neighbor’s farm, tourists can see them there”.*

A few respondents stated that the costs of keeping animals were too high as the reason for not keeping animals on their farm, and their production was described as unprofitable.

*Respondent 5: “Keeping animals is an additional cost, production is unprofitable.”*

The above responses from farmers indicate that they are aware of the limitations of keeping animals on the farm and providing them with good conditions and welfare. Perhaps these responses may indicate that if there was more interest from tourists, farmers would overcome the difficulties of not keeping their animals on their farm.

## 4. Discussion

The obtained results have practical and important consequences for agritourism owners. Our research shows that farmers consider animals an important element of their agritourism offering. This is confirmed by studies [73,74] that suggest that animals are frequently the key to attracting tourists to spend holidays in the countryside on an agritourism farm. 

The profitability of agritourism is an important factor from the point of view of the development of a tourist activity. The share of income from agritourism activities is important, which shows the development of agritourism as a source of additional income for the farming family [75]. It is very important to measure income from accommodations, food, and additional services: for example, by offering souvenirs to be purchased by tourists or agricultural products to be bought on the farm (fruits, vegetables, honey, milk, etc.), bike rental, guide services, horseback riding, etc. [75,76]. Studies cited by other authors show that agritourism has a significant and positive impact on farm profitability [43,77]. The value of income from agritourism varied and depended on the size of the facility, the agritourism offering, and the number of visiting tourists [75].

Livestock is an integral part of a sustainable farm [78]. The use of different animal species enables biodiversity conservation on the farm, expressed not only by the number of animal species but also by the diversity of crops necessary both for humans and animals, which simultaneously ensures maintenance of soil fertility [79]. 

Keeping animals can have long-term effects on the environment and can contribute to the preservation of characteristic plant species not found in neighbouring habitats [80]. Animals also contribute to the protection of the landscape, maintaining valuable plant communities in their natural state—an example may be the protection of xerothermic grasslands in western Poland, where sheep are used for this purpose [81].

In addition to providing a variety of food products and clothing products, the animals produce manure, which is a valuable natural fertiliser that enables a steady turnover of organic matter on the farm. Natural fertilisation is part of both sustainable farming and organic farming [82].

Large-scale farms, which also use large-scale animal husbandry, are widely used today [83]. The use of large-scale animal husbandry is justified for reasons of production cost reduction; however, it is frequently associated with the production of food products that do not have specific qualities. Animal husbandry in sustainable farms or organic farms certainly has good prospects because of the preservation of on-farm cycles (soil-plant-animal-soil), contribution to biodiversity conservation, and production of high-quality food products [3,84].

The grazing of livestock may affect wild species. It was found that the presence of cows on the pasture significantly influences the feeding efficiency of the white stork (*Ciconia ciconia*), and this may, in turn, contribute to increasing the population of the species [85]. Additionally, the presence of storks may be attractive to tourists [86].

Animals may be unnecessary on large-scale farms using mineral fertilisers, but on small farms concerned with sustainability and biodiversity, they are important because some farms may use their own manure as fertiliser.

In the case of agritourism farms, small-scale animal husbandry also leads to better interactions between visitors and animals. The guests, in addition to gaining knowledge concerning the functioning of the farm, also perform a sort of control function, especially in terms of animal husbandry conditions.

Animal husbandry in agritourism farms provides various opportunities for interacting with animals, from simple observation through participation in daily activities related to feeding, grooming, and herding to animal therapy, and finally to processing of animal food raw materials [87,88]. Agritourism farm owners often select animal species and breeds that are resistant to less favourable conditions for animal welfare, are resistant to diseases, and have interesting traits [89].

Maintaining high biodiversity in agricultural farms and, particularly, in agritourism farms is important, and organic agritourism farms should contribute not only to making the offer more attractive to guests but also to protecting and popularising the husbandry of local animal breeds and plant varieties that may become extinct in the current economic reality due to their frequently low productivity [90]. At the same time, animals should be treated as a cultural heritage element with great socio-cultural significance and an ever-increasing educational function [91], which generates intangible benefits associated with the region [92]. 

According to the farmers participating in our survey, an interesting measure from the point of view of guests visiting agritourism operators would be to introduce exotic animals to the livestock (e.g., alpacas, llamas). However, when it comes to maintaining local biodiversity and agritourism objectives, keeping exotic animals on farms seems questionable [93]. Exotic animals are certainly a factor that increases the attractiveness of the farm’s offering; however, agritourism activities carried out on sustainable farms, where traditional crops are grown and animals typical of the region are kept, are of great importance to become an opportunity for guests to learn about the agricultural culture of the region [80,85,92].

The native breeds of livestock, as well as native varieties of crops, are part of the national heritage [79]. The existence of such breeds proves the ability to carry out husbandry work aimed at creating animals that are best adapted to local environmental conditions (climate, fodder), which can demonstrate, frequently in difficult conditions, longevity, good parameters of reproductive traits, and production of food raw materials (meat, milk, eggs) or clothing raw materials (wool, leather) of high utility values [94,95,96]. Keeping native animal breeds is especially valuable in agritourism due to their adaptation to local environmental conditions, disease resistance, health, and low feed requirements [97]. The results of our research indicate that native breeds of livestock are not very common in agritourism. Research by other authors shows that they perform important popularizing, promotional, and educational functions, and tourists are eager to photograph them, like to observe them, and believe that their presence has a positive effect on the landscape [98,99,100].

Tourism needs are evolving, and tourists are looking for a multi-sensory rural experience [101]. The agritourism farms that offer attractions based on livestock are part of this trend. While there is not much literature concerning animal welfare in tourism or entertainment, there are articles analysing animal welfare in other sectors [102]. However, the concern for animal welfare contributes to the profitability of tourism [103]. 

In terms of the appreciation of farm animal welfare, it varies depending on producers and consumers. Consumers often focus on naturalness and its relation to animal welfare, while producers often focus on meeting basic health requirements [104]. Animals have a wide range of needs that are a consequence of many functional systems that make their life possible [78]. Animal welfare can be defined as “how well an animal manages to live under the conditions in which it exists” [105]. Healthy livestock is the most productive and economically viable, achieving increased growth rate, milk yield, or egg production per unit of input, as well as improved fertility, longer vitality, and better carcass quality [106].

The role of farmers should be emphasised; they are a sort of guide playing an important role as facilitators helping tourists to overcome environmental challenges and better understand the animal world [107].

## 5. Conclusions

This study makes a significant theoretical contribution to understanding the importance of livestock in building an agritourism product. 

A large part of the surveyed agritourism suppliers (57.3%) had animals, which are both an immanent feature of the agricultural holding and the agritourism business. In this case, there is a clear relationship related to the size of the land owned, which is important for having adequate space for these animals. Our research has shown that keeping animals in agritourism farms is an attraction for tourists visiting them.

Keeping livestock provided an attraction for tourists using the offered services, but it also provided a source of income from the sale of those animals. This is confirmed by the share of agricultural income in total income, which was higher for agritourism operators who owned animals than for those who did not own animals. Plus, it is also confirmed by a higher share of income from agritourism in the group of farms with animals.

The surveyed facilities usually keep several species of animals. Their selection is purposeful because these animals are not only key to increasing tourist attractiveness, but they also provide a source of meat, products, and preserves. 

The biodiversity conservation in agricultural holdings, especially in agritourism farms, should, on the one hand, contribute to the protection and popularisation of husbandry of local animal breeds and varieties of native plants and, on the other hand, make the offering more attractive for guests. 

This study does not exhaust the problem of keeping animals in agritourism farms. Therefore, it is important that such research is continued in the future, taking into account the opinions of various players in the agritourism market, both farmers and tourists. The survey used a questionnaire to obtain farmers’ opinions on keeping farm animals on agritourism farms. We believe that the research can be extended to include in-depth interviews with farmers running agritourism farms and keeping animals and those who stopped keeping animals completely. This would enable a better understanding of the importance of animals in creating a farm tourism product and the problems that arise for farmers from combining the two businesses.

Future research should aim to gain knowledge concerning the preferences of potential agritourists regarding which livestock they would like to encounter during their agritourism trips. In addition, further research is needed among farmers running agritourism activities in various regions of Poland and other European Union countries and the comparison of these results.

## Figures and Tables

**Table 1:** Characteristics of the surveyed agritourism operators.

Specification	Agritourism Farms with Animals (*n* = 142)	Animal-Free Agritourism Farms (*n* = 106)
Agricultural holding area [ha]		
Mean	17.6	16.0
Median	12.0	7.0
Maximum value	150.0	200.0
Share of income from agricultural activity in total income		
Mean	47.4%	42.9%
Share of income from agritourism activity in total income		
Mean	29.3%	25.6%
Share of other income in total income		
Mean	47.6%	67.9%

Source: own studies.

**Table 2:** Farm animals as an element of an agritourism product.

Structure of the Agritourism Product	Place and Role of Animals
the core of the product	the need for relations, being with animals, the need to know farm animals, need to touch
the base product	culinary products offered on the farm for tourists, the ability to purchase products, watching, feeding the animals, horse rides, tours with pets
the product extended	the opportunity to learn how to prepare a local culinary product, participation in the handling of animals, a small zoo, animal therapies

## Data Availability

All the data discussed in the manuscript are present within the text and tables.

## Supplementary Materials

The following are available online at https://www.mdpi.com/article/10.3390/ani11082357/s1, Questionnaires used for telephone interviews called CATI (computer assisted telephone interviews).

## References

[1] Zacharek N. (2017). Zwierzę domowe jako członek rodziny w XIX i XXI wieku. Tematy Z Szewskiej.

[2] Tryjanowski P., Hartel T., Baldi A., Szymański P., Tobółka M., Herzon I., Goławski A., Hromada M., Jerzak L., Kujawa K. (2011). Conservation of farmland birds faces different challenges in Western and Central-Eastern Europe. Acta Ornithol..

[3] Cassandro M. (2014). Extensive animal production and its added value in production and environmental chains: A dairy cattle study. Anim. Prod. Rev..

[4] Dashper K. (2018). Moving beyond anthropocentrism in leisure research: Multispecies perspectives. Ann. Leis. Res..

[5] Danby P., Dashper K., Finkel R. (2019). Multispecies leisure: Human-animal interactions in leisure landscapes. Leis. Stud.

[6] https://brainybackpackers.com/unethical-animal-tourism/.

[7] Higginbottom K., Higginbottom K. (2004). Wildlife Tourism: An Introduction. Wildlife Tourism: Impacts, Management and Planning.

[8] Moorhouse T., D’Cruze N.C., Macdonald D.W. (2017). Unethical use of wildlife in tourism: What’s the problem, who is responsible, and what can be done?. J. Sustain. Tour..

[9] Malchrowicz-Mośko E., Munsters W., Korzeniewska-Nowakowska P., Gravelle F. (2020). Kontrowersyjna turystyka z wykorzystaniem zwierząt w perspektywie kulturowej. Turyzm.

[10] Moorhouse T.P., Dahlsjö C., Baker S., D’Cruze N., Macdonald D. (2015). The customer isn’t always right: Conservation and ani mal welfare implications of the increasing demand for wildlife tourism. PLoS ONE.

[11] McAfee K. (1999). Selling nature to save it? Biodiversity and green developmentalism. Environ. Plan. D Soc. Space.

[12] Belicia T.X.Y., Islam M.S. (2018). Towards a Decommodified Wildlife Tourism: Why Market Environmentalism Is Not Enough for Conservation. Societies.

[13] Newsome D., Dowling R.K., Moore S.A. (2005). Wildlife Tourism.

[14] Harris R.B., Cooney R., Leader-Williams N. (2013). Application of the anthropogenic Allee effect model to trophy hunting as a conservation tool. Conserv. Biol..

[15] Animals in Tourism 2017. https://www.tourismconcern.org.uk/wp-content/uploads/2018/03/Animals-in-Tourism-lWeb-FINAL-1.pdf.

[16] Winter C. (2020). A review of research into animal ethics in tourism: Launching the annals of tourism research curated collection on animal ethics in tourism. Ann. Tour. Res..

[17] Essen E., Lindsjö J., Berg C. (2020). Instagranimal: AnimalWelfare and Animal Ethics Challenges of Animal-Based Tourism. Animals.

[18] Lubbe B.A., du Preez E.A., Douglas A., Fairer-Wessels F. (2019). The impact of rhino poaching on tourist experiences and future visitation to National Parks in South Africa. Curr. Issues Tour..

[19] Okello M.M. (2014). Economic Contribution, Challenges and Way Forward for Wildlife-Based Tourism Industry in Eastern African Countries. J. Tour. Hosp..

[20] Ryan C., Bolin V., Shirra L., Garrard P., Putsey J., Vines J., Hartny-Mills L. (2018). The development and value of whale-watch tourism in the west of Scotland. Tour. Mar. Environ..

[21] Istomina E.A., Luzhkova N.M., Khidekel V.V. (2016). Birdwatching Tourism Infrastructure Planning in the Ria Formosa Natural Park (Portugal). Geogr. Nat. Resour..

[22] Cardall T.Y., Rosen P. (2003). Grizzly Bear Attack. J. Emerg. Med..

[23] Snyder J., Stonehouse B., Snyder J., Stonehouse B. (2007). The growing significance of polar tourism. Prospects for Polar Tourism.

[24] Ivancso-Horváth Z., Ercsey I. (2016). A regional-based analysis of angling tourism. Int. Leis. Rev..

[25] Di Minin E., Leader-Williams N., Bradshaw C.J.A. (2016). Banning Trophy Hunting Will Exacerbate Biodiversity Loss. Trends Ecol. Evol..

[26] Buller H. (2004). Where the Wild Things Are: The Evolving Iconography of Rural Fauna. J. Rural Stud..

[27] Wearing S., Mcdonald M., Ponting J. (2005). Building a Decommodified Research Paradigm in Tourism: The Contribution of NGOs. J. Sustain. Tour..

[28] Buller H., Blokhuis H., Jensen P., Keeling L. (2018). Towards Farm Animal Welfare and Sustainability. Animals.

[29] Buller H., Roe E. (2013). Modifying and commodifying farm animal welfare: The economization of layer chickens. J. Rural Stud..

[30] Mkono M., Holder A. (2019). The future of animals in tourism recreation: Social media as spaces of collective moral reflexivity. Tour. Manag. Perspect..

[31] Young J., Carr N., Young J., Carr N. (2018). Introduction. Domestic Animals, Humans and Leisure: Rights, Welfare, and Wellbeing.

[32] Bertella G. (2014). The co-creation of animal-based tourism experiences. Tour. Recreat. Res..

[33] Danby P., Young J., Carr N. (2018). A post-humanistic insight into human-equine interactions and wellbeing within leisure and tourism. Domestic Animals, Humans, and Leisure.

[34] Koca T.T., Ataseven H. (2015). What is hippotherapy? The indications and effectiveness of hippotherapy. North Clin. Istanb..

[35] Glenk L.M. (2017). Current Perspectives on Therapy Dog Welfare in Animal-Assisted Interventions. Animals.

[36] Odendaal J.S.J. (2000). Animal-assisted therapy—Magic or medicine?. J. Psychosom. Res..

[37] Panzera M., Alberghina D., Statelli A. (2020). Ethological and Physiological Parameters Assessment in Donkeys Used in Animal Assisted Interventions. Animals.

[38] Grassberger M. (2013). Biotherapy—History, Principles and Practice: A Practical Quide to the Diagnosis and Treatment of Disease Using Living Organisms.

[39] Busby G., Rendle S. (2000). The transition from tourism on farms to farm tourism. Tour. Manag..

[40] Barlybaev A.A., Akhmetov V.Y., Nasyrov G.M. (2009). Tourism as a factor of rural economy diversification. Stud. Russ. Econ. Dev..

[41] Sharpley R., Vass A. (2006). Tourism, farming and diversification: An attitudinal study. Tour. Manag..

[42] Oppermann M. (1996). Rural tourism in southern Germany. Ann. Tour. Res..

[43] Jęczmyk A., Uglis J., Graja-Zwolińska S., Maćkowiak M., Spychała A., Sikora J. (2015). Research note: Economic benefits of agrotourism development in Poland: An empirical study. Tour. Econ..

[44] Majewski J., Lane B. (2001). Turystyka Wiejska i Rozwój Lokalny.

[45] Pavić L., Pažek K., Pavlovič M. (2018). Agritourism. Between agriculture and tourism; A review. 3rd International Thematic Monograph: Modern Management Tools and Economy of Tourism Sector in Present Era.

[46] Balińska A., Zawadka J. (2013). Znaczenie agroturystyki w rozwoju obszarów wiejskich. Zesz. Nauk. SGGW W Warszawie. Ekon. I Organ. Gospod. Żywnościowej.

[47] Sikora J. (2012). Agroturystyka. Przedsiębiorczość na Obszarach Wiejskich.

[48] Majewski J. (2000). Agroturystyka to Też Biznes.

[49] Sznajder M., Przezbórska L., Scrimgeour F. (2009). Agritourism.

[50] Roman M. (2015). Agritourism farms owners’ competence in running their economic activities. Polish J. Manag. Stud..

[51] Sikorska G., Kajszczak W. (2001). Kwatera Agroturystyczna. Praktyczny poradnik.

[52] Jęczmyk A., Uglis J., Zarzecka K., Kondracki S. (2014). Organizacyjno-prawne uwarunkowania rozwoju turystyki na obszarach wiejskich. Współczesne Dylematy Polskiego Rolnictwa III cz.

[53] Streifeneder T. (2016). Agriculture first: Assessing European policies and scientific typologies to define authentic agritourism and differentiate it from countryside tourism. Tour. Manag. Perspect..

[54] Cummins A.M., Widmar N.J.O., Croney C.C., Fulton J.R. (2016). Exploring Agritourism Experience and Perceptions of Pork Production. Agric. Sci..

[55] Hall P.K. (2019). Farm Animals and People: Liability Issues for Agritourism?. https://nationalaglawcenter.org/wp-content/uploads/assets/articles/Agritourism-series-Farm-Animals.pdf.

[56] Gandini G., Oldenbroek K., Oldenbroek K. (2007). Strategies for moving from conservation to utilization. Utilisation and Conservation of Farm Animal Genetic Resources.

[57] Kokocińska A., Kaleta T. (2016). The role of ethology in animal welfare. Sci. Ann. Pol. Soc. Anim. Prod..

[58] Zervas G., Tsiplakou E. (2011). The effect of feeding systems on the characteristic of products from small ruminants. Small Rumin. Res..

[59] Pinto-Correia T., Mascarenhas J. (1999). Contribution to the extensification/intensification debate: New trends in the Portugese montado. Landsc. Urban Plan..

[60] Kline C., Barbieri C., LaPan C. (2016). The influence of agritourism on niche meats loyalty and purchasing. J. Travel Res..

[61] Long L.M. (2003). Culinary Tourism.

[62] Hall C.M., Sharples L., Hall C.M., Sharples L., Mitchell R., Macionis N., Cambourne B. (2003). The consumption of experiences or the experience of consumption? An introduction to the tourism of taste. Food Tourism Around the World.

[63] Niedbała G., Jęczmyk A., Steppa R., Uglis J. (2020). Linking of Traditional Food and Tourism. The Best Pork of Wielkopolska—Culinary Tourist Trail: A Case Study. Sustainability.

[64] Ecker S., Clarke R., Cartwright S., Kancans R., Please P., Binks B. (2010). Drivers of Regional Agritourism and Food Tourism in Australia, Australian Goverment?. https://www.agriculture.gov.au/sites/default/files/abares/documents/agritourism-2010-report-11a.pdf.

[65] Ciolac R., Adamov T., Iancu T., Popescu G., Lile R., Rujescu C., Marin D. (2019). Agritourism-A Sustainable Development Factor for Improving the ‘Health’ of Rural Settlements. Case Study Apuseni Mt. Area Sustain..

[66] Sayre R., Henderson K., Kline C. (2018). The cow goes “moo”: Farm animal and interactions on Long Island’s North Fork. Animals, Food and Tourism.

[67] Hurst J.L., Niehm L.S. (2021). Tourism Shopping in Rural Markets: A Case Study in Rural Iowa. Int. J. Cult. Tour. Hosp. Res..

[68] Carr E.C.J., Warth A. (2001). The Use of the Telephone Interview for Research. NT Res..

[69] Belshaw Z., Robinson N.J., Dean R.S., Brennan M.L. (2018). Owners and Veterinary Surgeons in the United Kingdom Disagree about What Should Happen during a Small Animal Vaccination Consultation. Vet. Sci..

[70] Łuczka W., Kalinowski S. (2020). Barriers to the Development of Organic Farming: A Polish Case Study. Agriculture.

[71] Israel G.D. (1992). Determining Sample Size.

[72] Adam A.M. (2020). Sample Size Determination in Survey Research. J. Sci. Res. Rep..

[73] Kuźnicka E., Michałowski K., Balcerek M., Boruta A. (2015). Zwierzęta w gospodarstwie agroturystycznym jako element zwiększający atrakcyjność oferty. Wiad. Zootech..

[74] Uglis J., Jęczmyk A. (2017). Agroturystyka w Teorii i Praktyce.

[75] Roman M., Grudzień P. (2021). The Essence of Agritourism and Its Profitability during the Coronavirus (COVID-19) Pandemic. Agriculture.

[76] Hardesty S., Feenstra G., Visher D., Lerman T., Thilmany-McFadden D., Bauman A., Gillpatrick T., Rainbolt G.N. (2014). Values-based Supply Chains: Supporting Regional Food and Farms. Econ. Dev. Q.

[77] Lucha C., Ferreira G., Walker M., Groover G. (2016). Profitability of Virginia’s Agritourism Industry: A Regression Analysis. Agric. Resour. Econ. Rev..

[78] Broom D.M., Galindo F.A., Murgueitio E. (2013). Sustainable, efficient livestock production with high biodiversity and good welfare for animals. Proc. R. Soc. B.

[79] Dębska B., Długosz J., Piotrowska-Długosz A., Banach-Szott M. (2016). The impact of a bio-fertilizer on the soil organic matter status and carbon sequestration—results from a field-scale study. J. Soils Sediments.

[80] Kurek P., Steppa R., Grzywaczewski G., Tryjanowski P. (2016). The silence of the lambs? Plant diversity in abandoned sheep pens. Plant Soil Environ..

[81] Bernacka H., Niedźwiecki P., Kasperska D., Peter E. (2013). The behavior of breed sheep Wrzosówka on the xerothermic grasslands. Animal Prod. Rev..

[82] Dhiman V. (2020). Organic farming for sustainable environment: Review of existed policies and suggestions for improvement. Int. J. Res. Rev..

[83] Maurel M.C., Lacquement G. (2020). From the Large-Scale Farming to Agribusiness: Towards New Agricultural Capitalism in Central Europe. Village Agric..

[84] Barłowska J. (2011). The significance of native animal breeds in the production of traditional food and the transmission of tradition and culture of the region. Anim. Prod. Rev..

[85] Zbyryt A., Sparks T.H., Tryjanowski P. (2020). Foraging efficiency of white stork Ciconia ciconia significantly increases in pastures containing cows. Acta Oecol..

[86] Kronenberg J., Andersson E., Tryjanowski P. (2017). Connecting the social and the ecological in the focal species concept: Case study of White Stork. Nat. Conserv..

[87] Sczygiol M., Mrdalj V., Brković D. (2018). The combination of cheese dairy and agri-tourism as away of doing business in rural areas–Case study. Eur. J. Serv. Manag..

[88] Testa R., Galati A., Schifani G., Di Trapani A.M., Migliore G. (2019). Culinary Tourism Experiences in Agri-Tourism Destinations and Sustainable Consumption–Understanding Italian Tourists’ Motivations. Sustainability.

[89] Bernacka H., Umerska-Błażkiewicz M., Peter E. (2016). The role of sheep and goats in shaping the agricultural landscape. Anim. Prod. Rev..

[90] Biscarini F., Nicolazzi E.L., Stella A., Boettcher P.J., Gandini G. (2015). Challenges and opportunities in genetic improvement of local livestock breeds. Front. Genet..

[91] Hoffmann I., From T., Boerma D. (2014). Ecosystem Services Provided by Livestock Species and Breeds, with Special Consideration to the Contributions of Small-Scale Livestock Keepers and Pastoralists.

[92] Uglis J., Steppa R., Jęczmyk A., Zarzecka K., Kondracki S. (2014). Przyrodnicza różnorodność elementem uatrakcyjniającym ofertę agroturystyczną. Współczesne Dylematy Polskiego Rolnictwa III cz.

[93] Toland E., Bando M., Hamers M., Cadenas V., Laidlaw R., Martínez-Silvestre A., van der Wielen P. (2020). Turning Negatives into Positives for Pet Trading and Keeping: A Review of Positive Lists. Animals.

[94] Świtek S., Jankowiak Ł., Rosin Z.M., Sawinska Z., Steppa R., Takacs V., Zbyryt A., Tryjanowski P. (2017). How to Keep a High Level of Biodiversity on Farmland Area in Poland. Identif. Major Res. Probl. Village Agric..

[95] Migdał W., Golian J., Marcinčák S., Král M., Walczycka M., Domagała J., Najgebauer-Lejko D., Migdał Ł. (2020). The influence of the Wallachians on the pastoral culture and cuisine of the Carpatians. Anim. Prod. Rev..

[96] Niżnikowski R., Niemczyk J., Szymańska Ż. (2020). Analysis of the determinants and effects of restoration of the Kazimierzowska goat breed. Anim. Prod. Rev..

[97] Koperska N., Litwińczuk Z. (2014). Znaczenie rodzimych ras bydła w agroturystyce. Przegląd Hod..

[98] Sawa A., Bogucki M., Neja W., Jankowska M., Jaworska M., Ciszewski P. (2011). Znaczenie bydła w gospodarstwach agroturystycznych [Importance of cattle in agritourism farms]. Rocz. Nauk. Pol. Tow. Zootech..

[99] Sokół J.L. (2010). Zwierzęta w Gospodarstwach Agroturystycznych i Ich Otoczeniu.

[100] Sokół J.L. (2015). Rola zwierząt w tworzeniu produktu turystyki wiejskiej na przykładzie gospodarstw agroturystycznych północno-wschodniej Polski. Ekonomia Środowisko.

[101] Woods M. (2011). Rural.

[102] Fennell D.A. (2013). Tourism and Animal Welfare. Tour. Recreat. Res..

[103] Hall D., Brown F. (2006). Tourism and Welfare: Ethics, Responsibility and Sustained Well-Being.

[104] Buddle E.A., Bray H.J., Ankeny R.A. (2021). “Of course we care!”: A qualitative exploration of Australian livestock producers’ understandings of farm animal welfare issues. J. Rural Stud..

[105] Hewson C.J. (2003). What is animal welfare? Common definitions and their practical consequences. Can. Vet. J..

[106] Capper J.L., Williams P. (2019). Healthy Livestock Produce Sustainable Food.

[107] Bertella G. (2016). Experiencing nature in animal-based tourism. J. Outdoor Recreat. Tour..

