# Supplementary material for: Can Animals Be the Key to the Development of Tourism: A Case Study of Livestock in Agritourism
Source: Animals (Basel). 2021 Aug 9;11(8):2357. doi: 10.3390/ani11082357 (PMC8388776; doi:10.3390/ani11082357)
Supplement: Supplementary file 1 [file animals-11-02357-s001.zip › animals-1331644-supplementary.pdf]

Name of the agritourism farm ………………………………
DISTRICT ……………………………………………………
Location……………………….……………………………….

Do you operate a farm? YES NO

1. Please state the year you started your agritourism business ………………..
2. What is the number of guest rooms? ……………………………..
3. What is the number of beds?……………………………..
4. What is the number of people involved in guest services? …………………………….
5. What is the average number of tourists per year?…………………………….
6. What is the income structure? Please indicate the percentage to total 100%
   agricultural ………………… .. agritourism ……………………… other (what?) ……………………
7. What is the area of the farm in hectares?……………………………..

8. Are there any animals kept on the farm? (if not please go to question 10)

HORSES	NUMBER	BREED	demonstration	horse riding	hippotherapy	carriage and wagon rides	others

CATTLE	NUMBER	BREED	demonstration	processing of products	sale of products on farm	preparing meals for guests	others

PIGS	NUMBER	BREED	demonstration	processing of products	sale of products on farm	preparing meals for guests	others

SHEEP	NUMBER	BREED	demonstration	processing of products	sale of products on farm	preparing meals for guests	others

GOATS	NUMBER	BREED	demonstration	processing of products	sale of products on farm	preparing meals for guests	others

CHICKEN	NUMBER	BREED	demonstration	processing of products	sale of products on farm	preparing meals for guests	others

DUCKS	NUMBER	BREED	demonstration	processing of products	sale of products on farm	preparing meals for guests	others

GOOSE	NUMBER	BREED	demonstration	processing of products	sale of products on farm	preparing meals for guests	others

EXOTIC ANIMALS	NUMBER	BREED	demonstration	others

9. Are native animal breeds kept on the farm? specify the number

**CATTLE YES NO**			
POLISH BLACK AND WHITE	POLISH RED AND WHITE	POLISH RED	others

**PIGS YES NO**				
THE ZŁOTNICKA WHITE PIG	ZŁOTNICKA SPOTTED PIG	POLISH BIG WHITE PIG	POLISH WHITE FOLD PIG	others

**HORSES YES NO**				
POLISH PONY	HUTSUL	WIELKOPOLSKA HORSE	SILESIAN HORSE	others

**SHEEP YES NO**					
POLISH MERINO IN OLD TYPE	POLISH MERINO	ŚWINIARKA SHEEP	WIELKOPOLSKA SHEEP	HEATH SHEEP	others

**GOATS YES NO**						
CARPATHIAN	POLISH WHITE IMPROVED GOAT	POLISH COLORFUL IMPROVED GOAT	others			
**POULTRY**						
**CHICKEN YES NO**						
RED HEN	LEGHORN	SUSSEX	GREEN-LEGGED PARTRIDGE	YELLOW-LEGGED PARTRIDGE	others	
**DUCKS YES NO**						
PEKIN	others					
**GOOSE YES NO**						
BILGORAJ GOOSE	ZATORSKA GOOSE	HUNCHBACK GOOSE	KARTUSKA GOOSE	KIELCE GOOSE	SUBCARPATHIAN GOOSE	POMERANIAN GOOSE
RYPIŃSKA GOOSE	SUWAŁKI GOOSE	SLOVAK GOOSE	ROMANCE GOOSE	CUBAN GOOSE	LANDES GOOSE	others
**RABBITS YES NO**						
POPIELNO WHITE RABBIT	others					
**FISH**	**BEE**					

10. Are the guests interested in the issue of animals at the agritourism farm? (please select all appropriate answers)

a) questions at the time of booking
b) participation in show/demonstrations
c) feeding, watering, cleaning
d) milking
e) preserves / sale of products
f) others ……………………………………………………………………………………………

11. What are the reasons for the lack of animals on the agritourism farm? (please select all appropriate answers)

a) aesthetic considerations
b) lack of tourist interest
c) financial considerations
d) lack of knowledge, experience
e) no conditions for animal keeping
f) no tradition
g) others ……………………………………………………………………………………………

12. Can the introduction of native breeds make the offer of an agritourism farm more attractive?

a) definitely yes
b) rather yes
c) I do not know
d) rather not
e) definitely not

13. What species of animals can make the farm's offer more attractive?

………
………

14. What kind of animal activities do you think would be attractive for tourists visiting the farm?

………
………
